# Child Neurology at a Crossroads: Defining the Future of Training

**DOI:** 10.1212/NE9.0000000000200352

**Published:** 2026-08-17

**Authors:** Robert Thompson Stone, Margie A. Ream

**Affiliations:** 1 Department of Neurology, University of Rochester, NY; and; 2 Department of Pediatrics, The Ohio State University, Columbus.

Child neurology training stands at a crossroads as the Accreditation Council of Graduate Medical Education (ACGME) completes a comprehensive review of Child Neurology training requirements—a process that could reshape the future of the specialty.^1^ In the 6 decades since child neurology training emerged, the field has undergone extraordinary growth in scientific knowledge, patient volume and complexity, subspecialization, and therapeutic innovation.^2,3^ However, training structure and content have remained relatively stagnant.

Central to the ongoing discussions are longstanding disagreements regarding time-based rotation requirements for adult neurology and pediatrics. Under the current 5-year model, less than half of clinical training occurs in child neurology-specific learning environments. Many educators question whether this balance remains optimal in an era defined by subspecialization, genomic medicine, and rapidly expanding molecular therapies.^4,5^ Others argue that including these requirements remains essential for foundational skills and understanding neurologic disease across the lifespan.^6^

In their recent article in *Neurology*® *Education*, Cohen et al.^7^ report the findings of a Child Neurology Society (CNS) Task Force convened to examine the future of child neurology residency training. The task force included 13 voting members, largely current and former residency program directors and departmental leaders, selected by the CNS. Using a modified Delphi process that combined surveys and structured discussions, the authors sought consensus, defined as ≥75% concordance, regarding current and potential future training requirements.

The group reached consensus in several important areas, including the definition of a child neurologist, institutional settings for residency training, and the importance of training in neurogenetics and molecular therapies. Formal consensus was not achieved on the questions that have historically generated the greatest debate: the duration and structure of adult neurology and pediatrics training.

Although formal consensus was not reached,

“the majority of Task Force members opinions aligned with…a majority of child neurology residency program directors as well as child neurologists in practice and in training [who] favor reducing the specific time requirements spent in adult training and…pediatrics.”

Participants emphasized the growing importance of incorporating more transition-of-care training into both child and adult neurology residencies. The authors concluded that the curricular requirements should be “potentially modified in accordance with the evolving educational needs of child neurologists.” They emphasize that foundational knowledge continues to be essential, and that future discussions need to engage a broad group of stakeholders.

A major strength of the study was also one of its principal limitations. There is inherent value in discussions among a small group of well-established departmental leaders and educators who bring valuable perspectives on training, accreditation, and institutional considerations. However, important voices were minimally represented, including trainees and junior faculty who completed training more recently, both of whom may be less committed to traditional approaches. Other limitations to the strength of the conclusions include low initial survey response rate, small panel size, and lack of more rigorous methodology. The article reads more as a perspective than a formal research study; consequently, findings from consensus studies using more robust methods may be more reliable.^8,9^

The authors note that major changes to child neurology training create “a conundrum, as neither time nor funds ever seem sufficient to impart the totality of pertinent knowledge.” However, residency is not intended to provide complete knowledge required for a career. Rather, it should establish a strong foundation in core principles, provide broad child neurology exposure, and impart the skills needed for lifelong learning. A growing body of evidence and expert opinion suggests that reductions in adult neurology and pediatrics requirements may be possible while preserving these goals, making it difficult to justify disproportionate training burdens on child neurologists.^8,9^

Cohen et al. highlight the opportunity for flexibility where “some programs may have…less of a need for the trainees to rotate on adult neurology.” The exact clinical context in which foundational knowledge is best acquired may vary by institution. Time-based requirements can interfere with the most efficient utilization of these resources during residency, while flexibility will likely be beneficial in future ACGME program requirements. For example, there could be a range of allowable adult neurology time (e.g., 6–12 months), depending on pediatric patient volumes and subspecialist availability. However, such flexibility may mean residency applicants must choose program structure without a solid understanding of how different experiences will impact their future interests and careers.

Children with complex neurologic disorders are increasingly surviving into adulthood, underscoring the importance of effective transitions of care.^10^ The success of emerging therapies will amplify this issue. Historically, transitions training has been viewed as a child neurology concern; however, the growing population of adults living with childhood-onset and neurogenetic disorders may create an even greater need for changes in adult neurology training. Correcting the longstanding imbalance in cross-training between adult and child neurology represents an opportunity for meaningful reform.

Cohen et al. and others have highlighted the potential financial and credentialing challenges associated with major changes to child neurology training.^3,7^ Any changes to graduate medical education can have financial impacts, which will vary by institution because of variability in funding structures. However, decisions about training requirements should be driven primarily by the educational needs of child neurologists. Funding models and credentialing structures will ultimately need to adapt accordingly.

Throughout their article, Cohen et al. alternate between describing child neurology as a specialty and a subspecialty. This seemingly minor distinction reflects the field's identity crisis. The ACGME recognizes child neurology as a primary specialty. As the field looks toward the future, perhaps child neurologists no longer need to vacillate between being pediatricians who love neurology or neurologists who care for children. Perhaps they can simply be trained as child neurologists.
